# Specific urban units identified in tuberculosis epidemic using a geographical detector in Guangzhou, China

**DOI:** 10.1186/s40249-022-00967-z

**Published:** 2022-04-15

**Authors:** Hongyan Ren, Weili Lu, Xueqiu Li, Hongcheng Shen

**Affiliations:** 1 State Key Laboratory of Resources and Environmental Information System, Institute of Geographic Sciences and Natural Resources Research, Chinese Academy of Sciences, Beijing, 100101 China; 2 College of Resources and Environment, University of Chinese Academy of Sciences, Beijing, 100190 China; 3 Guangzhou Chest Hospital, Guangzhou, 510000 China

**Keywords:** Tuberculosis, Geographical detector, Specific urban units, Pairwise interaction, Guangzhou, China

## Abstract

**Background:**

A remarkable drop in tuberculosis (TB) incidence has been achieved in China, although in 2019 it was still considered the second most communicable disease. However, TB’s spatial features and risk factors in urban areas remain poorly understood. This study aims to identify the spatial differentiations and potential influencing factors of TB in highly urbanized regions on a fine scale.

**Methods:**

This study included 18 socioeconomic and environmental variables in the four central districts of Guangzhou, China. TB case data obtained from the Guangzhou Institute of Tuberculosis Control and Prevention. Before using Pearson correlation and a geographical detector (GD) to identify potential influencing factors, we conducted a global spatial autocorrelation analysis to select an appropriate spatial scales.

**Results:**

Owing to its strong spatial autocorrelation (Moran’s *I* = 0.33, *Z* = 4.71), the 2 km × 2 km grid was selected as the spatial scale. At this level, TB incidence was closely associated with most socioeconomic variables (0.31 < *r* < 0.76, *P* < 0.01). Of five environmental factors, only the concentration of fine particulate matter displayed significant correlation (*r* = 0.21, *P* < 0.05). Similarly, in terms of q values derived from the GD, socioeconomic variables had stronger explanatory abilities (0.08 < *q* < 0.57) for the spatial differentiation of the 2017 incidence of TB than environmental variables (0.06 < *q* < 0.27). Moreover, a much larger proportion (0.16 < *q* < 0.89) of the spatial differentiation was interpreted by pairwise interactions, especially those (0.60 < *q* < 0.89) related to the 2016 incidence of TB, officially appointed medical institutions, bus stops, and road density.

**Conclusions:**

The spatial heterogeneity of the 2017 incidence of TB in the study area was considerably influenced by several socioeconomic and environmental factors and their pairwise interactions on a fine scale. We suggest that more attention should be paid to the units with pairwise interacting factors in Guangzhou. Our study provides helpful clues for local authorities implementing more effective intervention measures to reduce TB incidence in China’s municipal areas, which are featured by both a high degree of urbanization and a high incidence of TB.

**Graphical Abstract:**

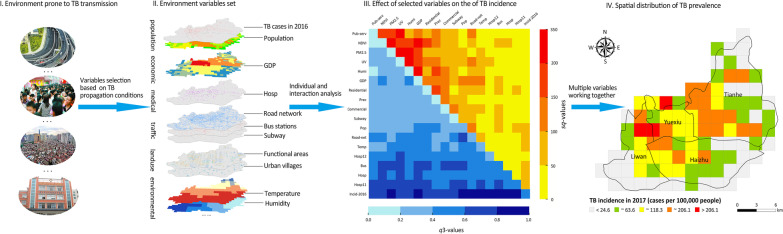

**Supplementary Information:**

The online version contains supplementary material available at 10.1186/s40249-022-00967-z.

## Background

Tuberculosis (TB) is a communicable disease that was the leading cause of death from a single infectious agent worldwide until the coronavirus disease 2019 pandemic [1]. TB is caused by the bacillus *Mycobacterium tuberculosis*, which is spread when people who are sick expel bacteria into the air (e.g., by coughing or talking), and typically attacks the lungs (pulmonary TB) [1–3]. Owing to the progress made in providing essential TB services by health authorities in different countries worldwide, a large global drop in the number of newly diagnosed TB cases has been achieved [1]. Over the last 15 years, the incidence of TB has declined to 55.55 per 100,000 inhabitants in China; nevertheless, it was still the second most communicable disease in China in 2019 [4]. Among China’s southeast coastal provinces, which have a relatively low TB incidence and better socioeconomic development than China’s central and western regions, Guangdong presented the highest incidence, which is somewhat surprising based on its socioeconomic situation. This puzzle has increasingly attracted the attention of researchers [5].

Scholars from around the world have conducted considerable research into TB epidemics, including the risk factors affecting its transmission and the corresponding prevention and control measures [6–12]. These studies have shown that the survival, suspension, and spread of *M. tuberculosis* expelled by infected people were often prolonged and promoted by environmental factors (e.g., high temperature, appropriate humidity, and a certain concentration of airborne particulate matter), while the dispersal of its carriers (e.g., saliva and particulate matter) was inhibited by frequent precipitation and favorable vegetation coverage [13–15]. Meanwhile, the exposure and infection probability of susceptible populations, as well as the diagnosis and treatment of TB cases, are heavily influenced by a series of social and economic factors (e.g., higher population density, more frequent population flow, uneven household income, scarce medical resources, and a well-developed public transportation system) [9, 16, 17]. However, possible interactions or combinations among these potential influencing factors, the spaces where they tend to happen, and their relationships with the incidence of TB remain underexplored.

Moreover, many epidemiological studies have been conducted to identify the dominant influencing factors in some endemic areas at various spatial scales, including the country, province, city, county or district, township or street, village, and even regular grid, which was meaningful for health authorities designing and implementing targeted interventions to reduce the incidence of TB [6, 7, 9, 12, 16–20]. However, the key factors identified as influencing TB epidemics in the above investigations were different due to the different spatial scales used. There have been a number of studies on the optimal choice of scale, especially for regular grids [21–23]. To some extent, small spatial scales are often the final units where prevention and control measures can produce practical effects, and more research into the factors influencing the incidence and prevention of TB is required on a fine spatial scale, especially within a city or its internal areas [24, 25].

Therefore, this study was conducted to characterize the spatial patterns of the 2017 incidence of TB across the central areas of Guangzhou through spatial autocorrelation analysis, and a geographical detector (GD) was used to further identify specific urban units with potential socioeconomic and environmental factors affecting this disease’s spread on a fine scale. The aim here was to provide effective guidance for relevant government departments designing and implementing targeted prevention and control measures to reduce the incidence of this disease in highly urbanized regions with severe TB epidemics.

## Methods

### Study area

**Fig. 1:**
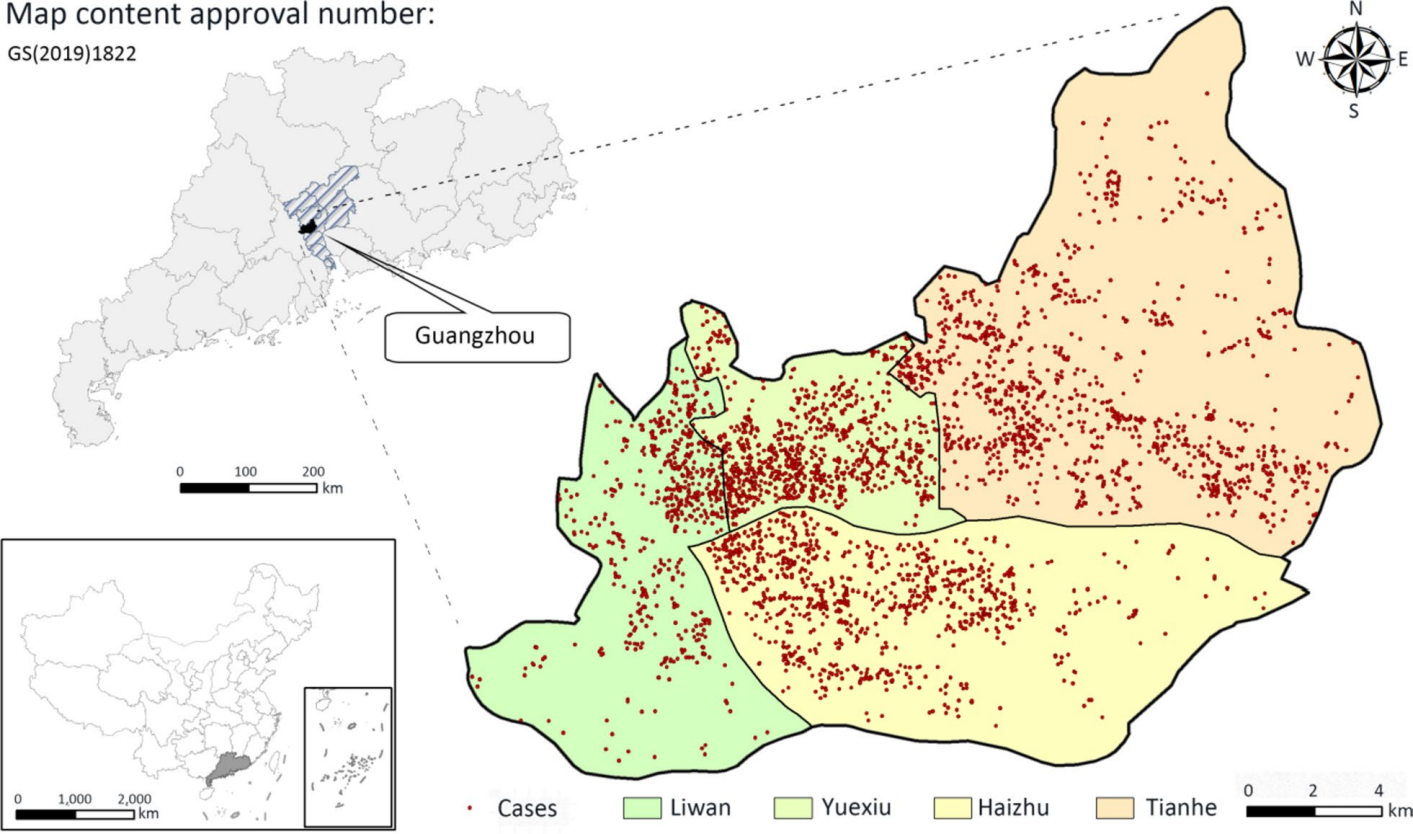
Illustration of study area with the spatial distribution of tuberculosis cases in 2017

Guangzhou City is a typical representative of China's megacities with more frequent population flows, more efficient and complex functional zones, more plentiful and fragmentized types of land uses, and more places or sites featured by variable microclimates [26]. The characteristics of its subtropical monsoon climate are obvious: warm and rainy, enough light and heat, an annual average temperature of 21–23 ℃, and an average annual precipitation of 1800 mm. As the most important districts in Guangzhou City, the four central districts (i.e., Yuexiu, Haizhu, Tianhe, and Liwan) are featured by their higher population density, vigorous economic activities, more frequent population flows, comprehensive public facilities, and convenient public transportation [27], by which we consider them as the study area (Fig. 1).

### Data collection

The TB epidemic data were obtained from Guangzhou Institute of Tuberculosis Control and Prevention, and included TB cases data from 2016 and 2017 (taking the newly diagnosed TB cases reported in 2017 as the dependent variable and existing cases reported in 2016 as a potential influencing factor). Relevant information included age, sex, permanent residence address, and occupation, as well as time of disease onset and diagnosis. Permanent residence address data was used in conjunction with geocoding (restapi.amap.com/v3/geocode) and coordinate deviation correction to produce cases data for a spatial point layer (Fig. 1) using ArcGIS 10.3 (ESRI, Redlands, CA, USA) software, in which the ratio of the number of TB cases to the total population in 2015 was calculated on a fine scale to indicate the TB incidence rates across the central districts. In 2017, the incidence of TB in Guangzhou was relatively high, with a total of 14,100 newly diagnosed cases, of which 4,313 were from the study area, accounting for 30.6% of the total, while this area only accounts for 4.5% of the entire area of Guangzhou.

According to previous studies [2, 7, 9, 12, 15–17, 28, 29], we gathered 18 potential factors from various data sources and then categorized them into two groups, listed in Table 1. Among 11 socioeconomic factors, the population-related factors included the incidence of TB in the previous year and the 1 km × 1 km gridded population density. The economic situation was analyzed based on the 1 km × 1 km gridded gross domestic product (GDP) per capita, and information with respect to officially appointed medical institutions included the medical resources that have been officially certified by local health departments to supply local residents with professional health services and to facilitate reimbursement of health service expenses to the patients. The road network, bus stops, and subway stations were selected to represent the condition of the public transportation system. With regard to potential influences of land use, the percentages of four typical kinds of land use in the central area—residential, commercial service, public service, and urban villages—were also calculated in this study. In addition to the above socioeconomic variables, five environmental conditions in 2017, including monthly mean values of the normalized difference vegetation index and the fine particulate matter concentration, as well as the average climatic conditions (temperature, precipitation, and humidity) from March to June, were simultaneously considered as potential variables influencing the prevalence of TB.

**Table 1:** Data collection and resources in this study

Data group	Selected variables by previous studies	Data type	Source
Socioeconomic factor	TB incidence rates in 2016 (Incid-2016) [9, 16]	Vector	Guangzhou Center for Disease Control and Prevention
	Population density (Pop) in 2015 [9, 12, 15, 16]	Raster (1 km)	Resource and Environment Science and data center (https://www.resdc.cn/)
	Gross domestic product per capita (GDP) in 2015 [7, 15]	Raster (1 km)	
	Officially appointed medical institutions (Hosp) [2, 28]	Vector	Guangzhou Municipal People's Government (http://www.gz.gov.cn/)
	Density of road network (Road_net) [17]	Vector	Open Street Map (http://download.geofabrik.de/)
	Numbers of subway stations (Subway)[17]	Vector	
	Counts of bus stops (Bus) [17]	Vector	
	Percentage of residential land (Residential) [16]	Vector	Tsinghua University (http://data.ess.tsinghua.edu.cn) [30]
	Percentage of commercial service land (Commercial) [16]	Vector	
	Percentage of land for public services (Pub-serv) [16]	Vector	
	Percentage of urban village area (UV) [29]	Vector	Our earlier study [31]
Environmental condition	Monthly average of the normalized difference vegetation index (NDVI) [15]	Raster (1 km)	MODIS (https://modis.gsfc.nasa.gov/)
	Monthly average of the fine particulate matter concentration (PM_2.5_) [15]	Raster (1 km)	Socioeconomic Data and Applications Center (https://sedac.ciesin.columbia.edu/data/sets/browse)
	Average temperature from March to June (Temp) [13, 15]	Raster (1 km)	China Meteorological Data Service Center (http://data.cma.cn/)
	Average precipitation from March to June (Prec) [13, 15]	Raster (1 km)	
	Average humidity from March to June (Humi) [13, 15]	Raster (1 km)	

For the data of officially appointed medical institutions, medical points that do not serve the community (only designated personnel) were removed according to their service recipients. The officially appointed medical institutions were then divided into outpatient (Hosp11), inpatient and outpatient (Hosp12) types according to the range of medical institution services.

To facilitate statistical and spatial analysis, the dependent variable (2017 incidence of TB) and 18 independent variables, with different data types (vectors) or diverse resolutions (raster) as given in Fig. S1 (Additional file 1), were summarized and aggregated into a uniform spatial scale by using the spatial join, zonal statistics, and field calculation tools in ArcGIS 10.3 software.

### Spatial scale

Owing to the constant changes of administrative divisions in China—in particular the districts, towns, streets, and villages—research units assigned by these divisions in relevant studies were likely to cause difficulties in conducting spatiotemporal analysis. To avoid this issue, replacing them with some regular grids is an appropriate solution [21]. In particular, these spatial grids are gradually considered as the final units where prevention and control measures can produce practical effects in urban regions [24, 25].

In this study, a series of regular grids (1 km × 1 km–5 km × 5 km) were constructed, by which the optimal grid scale characterizing the spatial pattern of TB epidemic was selected based on Moran 's *I* and *Z*-scores [32]. Moran 's *I* was calculated as follows.

1 $$\begin{array}{c}I=\frac{n\sum_{i=i}^{n}\sum_{j=1}^{n}{\omega }_{ij}\left({x}_{i}-\overline{x }\right)\left({x}_{j}-\overline{x }\right)}{\sum_{i=i}^{n}\sum_{j=1}^{n}{\omega }_{ij}\sum_{i=1}^{n}{\left({x}_{i}-\overline{x }\right)}^{2}}\end{array}$$

where *n* is the number of grids in the study area, ${x}_{i}$ and ${x}_{j}$ represent the TB incidence rates in grids i and j, respectively. ${\omega }_{ij}$ is the spatial weight. Global Moran 's *I* is generally tested by the *Z-*score/*P*-value, and the value varies from − 1 to 1. A higher Moran's *I* (larger *Z*-score and proper *P*-value) indicates greater similarity among attributes between adjacent spatial grids, which reveals that the TB epidemic is clustered in the region, whereas a low negative value indicates dissimilarity between adjacent grids and shows that the TB epidemic is discretely distributed in the region. In this study, Moran 's *I* and *Z*-scores of the TB incidence rates with different grid sizes were used to assess the optimal grid scales of the regional TB epidemic. Global Moran 's *I* was calculated using ArcGIS 10.3.

### Statistical analysis

The geographical detector is a statistical tool (http://geodetector.cn/) for detecting spatial heterogeneity and its determinants [33]. In this study, the GD was used to detect the influence of the socioeconomic and eco-environmental factors on the incidence of TB on an appropriate grid scale. The method assumes that if the selected factors are associated with the 2017 incidence of TB, they have a similar spatial distribution. This coupling is calculated as follows:

2 $$\begin{array}{c}q=1-\frac{1}{N{\sigma }^{2}}\sum_{h=1}^{L}{N}_{h}{\sigma }_{h}^{2}\end{array}$$

where *N* and *σ*^2^ are the total counts of grid units and the variance of the incidence of TB in 2017, respectively, and *h* = 1, 2, …, *L*, where *L* is the number of sub-areas of the study area divided by the detection factor X. The number of strata *L* might be 2–10 or more, according to prior knowledge or a classification algorithm. Here, *q* measures the association between the 2017 incidence of TB and the detection factor *X*, both linearly and nonlinearly, meaning that the explanatory power or ability of the detection factor *X* for the spatial heterogeneity of the gridded incidence of TB in 2017 is 100% × *q*, where *q* ∈ [0,1]. Note that *q* = 0 indicates that there is no coupling between the 2017 incidence of TB and *X*, while *q* = 1 indicates that this incidence is completely determined by *X*.

The interaction detection of the GD is also used to determine the explanatory ability of the interaction between any two factors for the spatial heterogeneity of the gridded incidence of TB in 2017. The interaction effects are judged by the following rules.

$$Enhance,nonlinear- :\mathrm{ q}\left({X}_{1}\cap {X}_{2}={X}_{3}\right)>q\left({X}_{1}\right)+q({X}_{2})$$

$$Independent :\mathrm{q}\left({X}_{1}\cap {X}_{2}={X}_{3}\right)=q\left({X}_{1}\right)+q({X}_{2})$$

$$Enhance, bi- : \mathrm{q}\left({X}_{1}\cap {X}_{2}={X}_{3}\right)>\mathrm{Max}(q\left({X}_{1}\right),q\left({X}_{2}\right))$$

$$Weaken, uni-:Min(q\left({X}_{1}\right),q({X}_{2}))<\mathrm{q}\left({X}_{1}\cap {X}_{2}={X}_{3}\right)<\mathrm{Max}(q\left({X}_{1}\right),q\left({X}_{2}\right))$$

$$Weaken, nonlinear:\mathrm{q}\left({X}_{1}\cap {X}_{2}={X}_{3}\right)<Min(q\left({X}_{1}\right),q\left({X}_{2}\right))$$

The enhancement effect of the interaction of variables is evaluated using the indicator *sq* with the following equation.

3 $$\begin{array}{c}sq=\frac{{q}{(X_3)}-\mathrm{Max}\left({q}{(X_1)},{q}{(X_2)}\right)}{\mathrm{Max}\left({q}{(X_1)},{q}{(X_2)}\right)}*100\%\end{array}$$

where ${X}_{3}={X}_{1}\cap {X}_{2}$ indicates the interaction of the detection factors X_1_ and X_2_$. q\left({X}_{1}\right)$,$q({X}_{2})$, $q\left({X}_{1}\cap {X}_{2}={X}_{3}\right)$ are the calculated *q* values of factors${X}_{1}$,${X}_{2}$, and${X}_{3}$. Min$q\left({X}_{1}\right)$,$q({X}_{2})$), Max ($q\left({X}_{1}\right)$,$q({X}_{2})$) denote the minimum and maximum values of *q* corresponding to ${X}_{1}$ and${X}_{2}$. The larger *sq* indicates that the greater the enhancement in the ability to explain the spatial heterogeneity of TB incidence when the two factors interact.

## Results

### Epidemiological characteristics

In 2017, 4,313 newly diagnosed TB cases were reported in Yuexiu, Tianhe, Haizhu, and Liwan, where the case density was 13.29 cases/km^2^, compared to only 1.95 cases/km^2^ in the whole city of Guangzhou. According to the proportion of TB patients’ occupation, age, and gender in the study area (Table 2), more than 72% of the total TB cases were reported among those with occupations of household/unemployed (33.1%), retired (23.9%), and commercial services (15.1%). Among the four age groups, the 19–45-year-old population accounted for the highest proportion, and the 0–18-year-old population had the lowest. The ratio of the number of male to female cases was about 7:3, which was consistent with the entire city of Guangzhou. These results show that the distribution of the TB epidemic in the four central districts was impacted by age, gender, and occupation.

**Table 2:** The age, and occupation distributions of tuberculosis cases in the four central districts

Occupation	Guangzhou City		Four central region		
	No. of cases	Proportion, %^†^	No. of cases	Proportion, %^†^	Proportion, %^‡^
Household and unemployment	4,176	29.6	1,428	33.1	34.2
Farmer	2,322	16.5	98	2.3	4.2
Retired	1,675	11.9	1,030	23.9	61.5
Worker	1,333	9.5	276	6.4	20.7
Business service	1,155	8.2	652	15.1	56.5
Unknown	1,122	8.0	182	4.2	16.2
Labor	627	4.4	26	0.6	4.2
Others	822	5.8	242	5.6	29.4
Student	491	3.5	212	4.9	43.2
Cadre	246	1.7	105	2.4	42.7
Catering service	131	0.9	62	1.4	47.3
	14,100	100.0	4,313	100.0	30.6*
*Age, years*					
0–18	380	2.7	125	2.9	32.9
19–45	6,751	47.9	1,933	44.8	28.6
46–60	3,519	25.0	1,066	24.7	30.3
> 60	3,450	24.4	1,189	27.6	34.5
	14,100	100.0	4,313	100.0	30.6*
*Gender*					
Male	9,967	70.7	2,976	69.0	29.9
Female	4,133	29.3	1,337	31.0	32.4
	1,4100	100.0	4,313	100.0	30.6*

Among the occupations, other occupations include teachers, fishermen, herders, etc., with less than 100 cases in the whole Guangzhou city. **† **Means the number of cases with this attribute as a percentage of the total number of cases in the study area. ‡ Represents the proportion of cases in the study area to the number of cases in the corresponding occupation or age group in Guangzhou. 30.6* Means the number of cases in the study area as a percentage of the total number of cases in the Guangzhou city

In addition to the above epidemiological characteristics, which were similar to those of the whole city of Guangzhou, several unique characteristics were also observed. The number of TB patients over 60 years old and the number of patients with the occupation of household/unemployed accounted for almost one-third of cases in the whole city. Meanwhile, about 40% of either the student or the cadre (and office clerk) TB cases in this city were reported within the four districts. Among the TB cases in the business and catering service (1,286 cases) of Guangzhou, more than half were located in the central districts (714 cases). In addition, the proportion of female cases in the central districts (31.0%) was slightly higher than the level of the whole city of Guangzhou (29.3%), while the proportion of male cases was slightly lower (69.0%). These analyses indicated that the incident of TB in the study area shared some features of the incidence of TB in the whole city, but also possessed its own characteristics.

According to the Moran’s *I* values derived from our global spatial autocorrelation analysis (Table 3), the 2017 incidence of TB in the central area was clearly spatially differentiated at various grid scales ranging from 1 km × 1 km to 5 km × 5 km. Among these, the 2 km × 2 km grid possessed the best ability to characterize the spatial distribution of the 2017 incidence of TB in the study area. Thus, the analyses that follow were conducted at this level.

**Table 3:** Global Moran’s *I* value of tuberculosis incidence at various grids in the study area

Grid scale	1 km	2 km	3 km	4 km	5 km
Moran’ *I*	0.25	**0.33**	0.26	0.00	0.31
*Z*-score	6.70	**4.71**	2.90	0.30	2.31
*P*-value	0.00	**0.00**	0.00	0.77	0.02

*P*-value < 0.01 or |*Z*-value > 2.58, *P*-value < 0.05 or |*Z*-value| > 1.96 and *P*-value < 0.1 or |*Z*-value| > 1.65 indicates this value is significant at the level of 0.01, 0.05, and 0.10

### Individual effects of selected variables

**Fig. 2:**
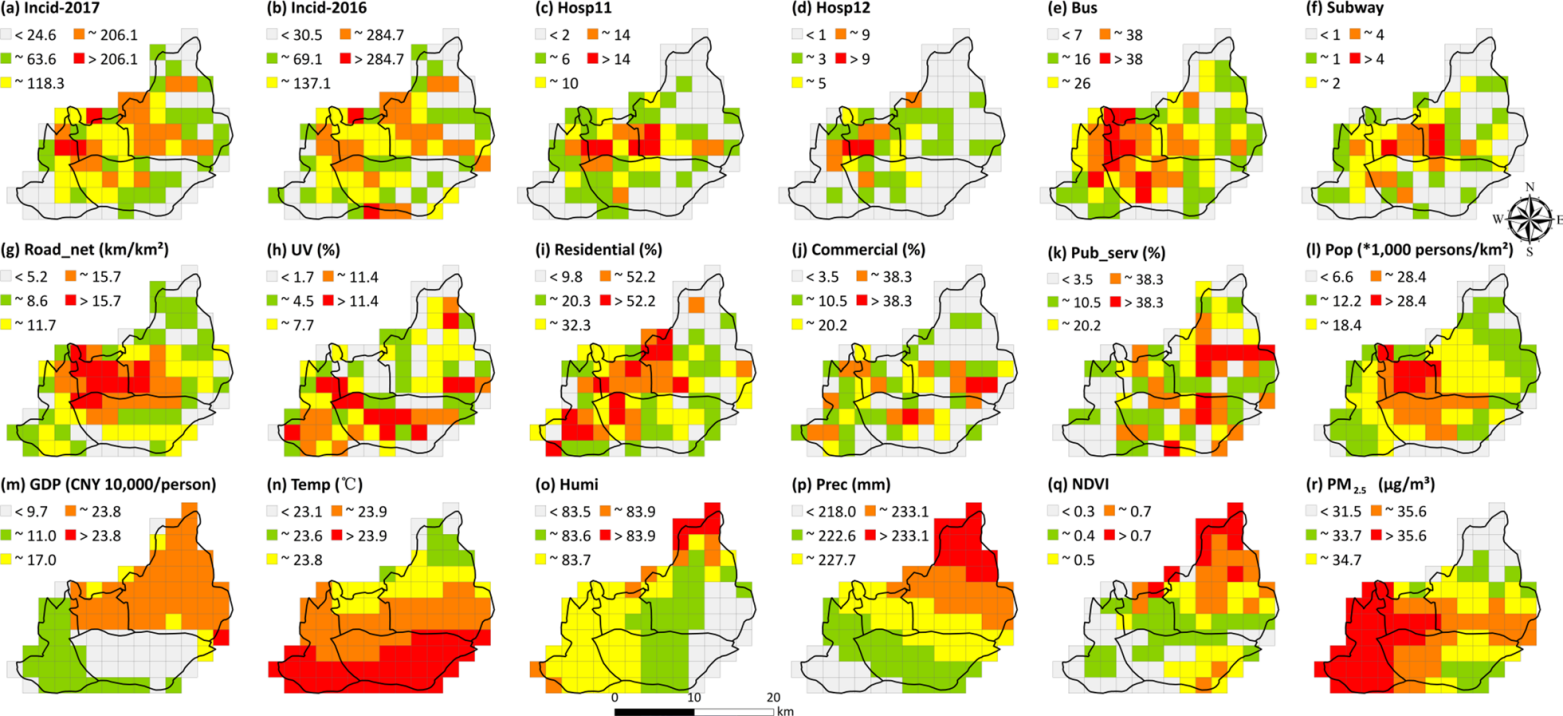
Spatial distribution of dependent variable (**a**) and included 18 independent variables (**b**–**q**) in this study. Due to the variable Hosp is equal to Hosp11 + Hosp12, it is not repeatedly shown here. The Map Content Approval Number: GS(2019)1822. ~ means greater than or equal to the previous number and less than or equal to the next number. *Incid-2017* TB incidence in 2017, *Incid-2016* TB incidence in 2016, *Pop* population density, *GDP* Gross National Product per capita, *Hosp* counts of officially appointed medical institutions, *Hosp11* outpatient hospitals of the officially appointed medical institutions, *Hosp12* inpatient and outpatient hospitals of the officially appointed medical institutions, *Road_net* road network density, *Subway* counts of subway stations, *Bus* counts of bus stops, *Residential* percentage of residential land area, *Commercial* percentage of commercial service land, *Pub-serv* percentage of land for public services, *UV* percentage of urban village area, *NDVI* annual monthly average normalized differential vegetation index, *PM*_*2.5*_ annual monthly average PM_2.5_ concentration, *Temp* average temperature from March to June, *Prec* average precipitation from March to June, *Humi* average humidity from March to June

The spatial distributions of 18 variables included in this study were clearly featured on the 2 km × 2 km grid scale (Fig. 2). Meanwhile, the grids with a high incidence of TB in 2017 were surrounded by several grids that had experienced a relatively high incidence of TB in 2016, had more bus stops or subway stations, had more officially appointed medical institutions, had a higher population density, had a higher PM_2.5_ concentration, and had lower NDVIs (Fig. 2). These results indicate that the spatial patterns of the gridded 2017 incidence of TB are likely associated with those of the 18 selected factors.

Meanwhile, the gridded incidence of TB in 2017 was closely associated with the majority of independent variables (Table 4). Among them, most of the socioeconomic factors presented significant positive relationships with this epidemic (0.37 < *r* < 0.76, *P* < 0.001), except for UV, GDP, and Pub-serv. In comparison with these socioeconomic factors, only one environmental variable (PM_2.5_ concentration) was closely correlated with the gridded incidence of TB in 2017 (*r* < 0.21, *P* < 0.001). As a whole, the 2017 incidence of TB in the four districts of Guangzhou tended to be more heavily affected by socioeconomic factors than by environmental conditions.

**Table 4:** Correlation coefficients between tuberculosis incidence and variables and the *q*-values derived from geographical detector analysis

Variable type	Variable name	*r*	*q* values
Socioeconomic variables	Incid-2016	0.76***	0.57***
	Hosp	0.67***	0.45***
	Hosp11	0.65***	0.49***
	Hosp12	0.54***	0.38***
	Bus	0.69***	0.44***
	Road-net	0.47***	0.26**
	Pop	0.31***	0.23***
	Subway	0.42***	0.19**
	Commercial	0.37***	0.16*
	Residential	0.37***	0.14**
	GDP	0.16	0.11*
	UV	0.17*	0.08
	Pub-serv	− 0.01	0.02
Environmental variables	Temp	0.01	0.27***
	Prec	0.06	0.15***
	Humi	− 0.07	0.10*
	PM_2.5_	0.21**	0.08
	NDVI	− 0.11	0.06

*r* is the Pearson correlation coefficient. ***, **, and * indicates this value is significant at the level of 0.01, 0.05, and 0.10. *Incid-2016 *TB incidence in 2016, *Pop* population density, *GDP* gross national product per capita, *Hosp* counts of officially appointed medical institutions, *Hosp11* outpatient hospitals of the officially appointed medical institutions; *Hosp12* inpatient and outpatient hospitals of the officially appointed medical institutions, *Road_net* road network density, *Subway* counts of subway stations, *Bus* counts of bus stops, *Residential* percentage of residential land area, *Commercial* percentage of commercial service land, *Pub-serv* percentage of land for public services, *UV* percentage of urban village area, *NDVI* annual monthly average normalized differential vegetation index, *PM*_*2.5*_ annual monthly average PM_2.5_ concentration, *Temp* average temperature from March to June, *Prec* average precipitation from March to June, *Humi* average humidity from March to June

In addition, the explanatory ability of each influencing factor, in terms of *q*-values as given in Table 4, was acquired using the GD. Among them, the majority of socioeconomic variables, excluding UV (*q*1 = 0.08, *P* > 0.10) and Pub-serv (*q*1 = 0.02, *P* > 0.10), possessed powerful explanatory abilities (0.11 < *q* < 0.57, *P* < 0.10) for the spatial differentiation of the gridded incidence of TB in 2017. In particular, three socioeconomic factors (i.e., the 2016 incidence of TB, the counts of officially appointed medical institutions, and the number of bus stops) accounted for about 44% of the spatial heterogeneity of the 2017 incidence of TB across the four central districts. In comparison, the environmental factors (e.g., the monthly averages of temperature, precipitation, and humidity) presented relatively lower explanatory abilities (0.10 < *q* < 0.27, *P* < 0.10), even though they were not closely associated with the 2017 incidence of TB. These results further illustrate that socioeconomic factors had greater impacts than environmental factors on the spatial heterogeneity of the gridded incidence of TB in 2017 in the central regions.

### Influences of pairwise interactions

**Fig. 3:**
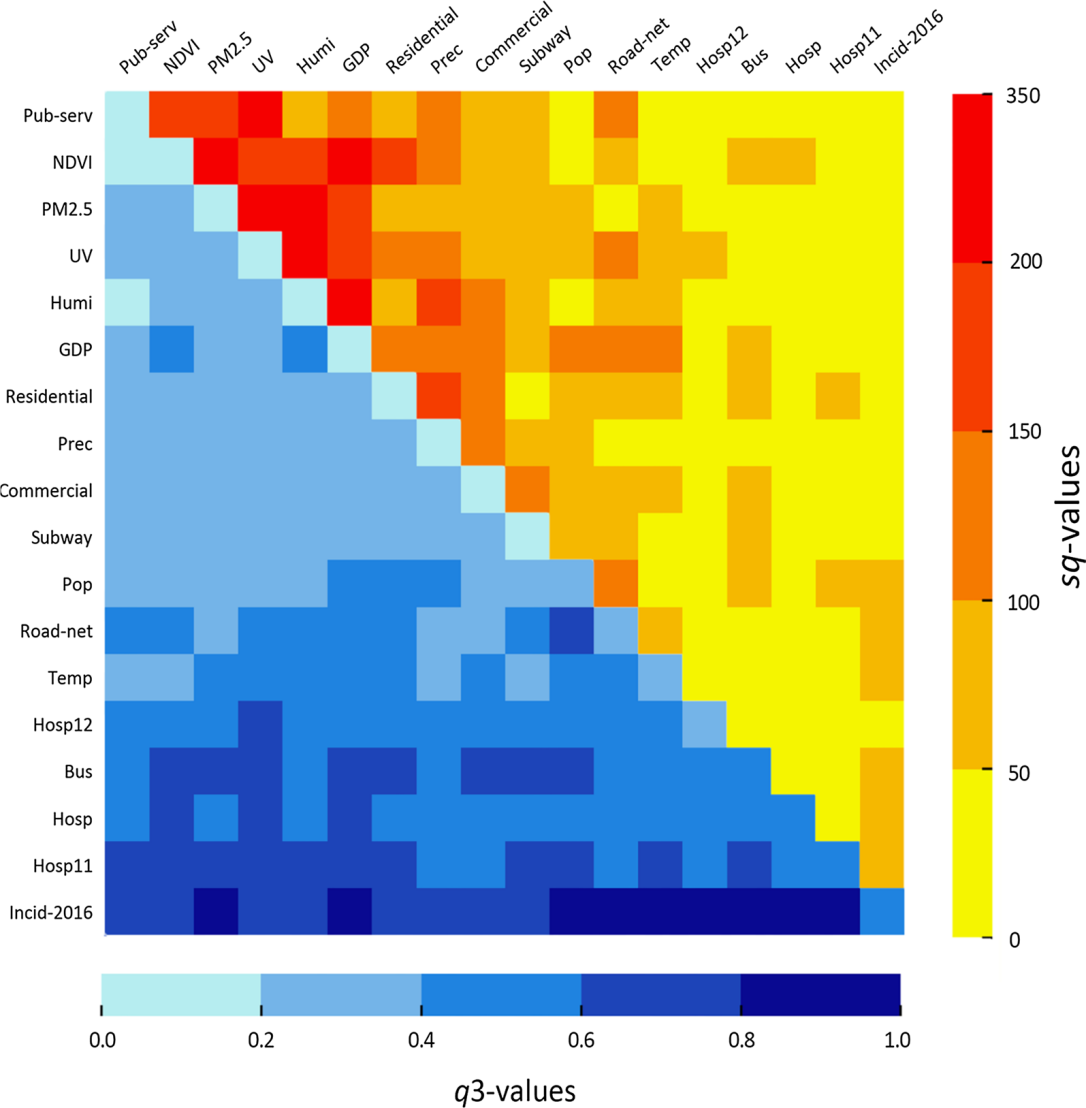
Illustration of the *q*3 (the lower left half) and *sq* (the higher right half) values for the pairwise interactions among the selected factors. *Incid-2016* TB incidence in 2016, *Pop* population density, *GDP* Gross National Product per capita, *Hosp* Counts of officially appointed medical institutions, *Hosp11* outpatient hospitals of the officially appointed medical institutions, *Hosp12* inpatient and outpatient hospitals of the officially appointed medical institutions, *Road_net* Road network density, *Subway* Counts of subway stations, *Bus* Counts of bus stops, *Residential* Percentage of residential land area, *Commercial* Percentage of commercial service land, *Pub-serv* Percentage of land for public services, *UV* Percentage of urban village area, *NDVI* Annual monthly average normalized differential vegetation index, *PM*_*2.5*_ Annual monthly average PM_2.5_ concentration, *Temp* Average temperature from March to June, *Prec* Average precipitation from March to June, *Humi* Average humidity from March to June

According to the *q*3 values varying from 0.16 to 0.89 (the lower left half in Fig. 3), the 153 pairwise interactions between the 18 individual variables presented much stronger abilities of interpreting the spatial differentiations of the gridded incidence of TB in 2017 than those of the 18 individuals alone (the diagonal grids from the left top to the right bottom in Fig. 3), resulting in obvious improvements termed by the *sq* values ranging from 7.3% to 311.6% (the upper right half in Fig. 3). Then, the 153 pairwise interactions could be accordingly divided into three groups: 68 pairs (*sq* < 50%), 49 pairs (50% < *sq* < 100%), and 36 pairs (*sq* > 100%), among which about 55.6% of the total pairs showed notable enhancements (*sq* > 50%). These results displayed that the pairwise interactions between selected potential influencing factors possessed much stronger explanatory abilities for the spatial differentiation of the gridded incidence of TB in 2017.

Furthermore, 153 pairwise interactions, in terms of the values of *q*3 and Maximum (*q*1, *q*2), were further classified into five groups (by *q*3 values) and three grades (by maximum values), yielding nine subgroups as given in Table 5. Among the 45 pairs within the first grade of Maximum (*q*1, *q*2), the majority (40 pairs, about 88.9%) presented moderately increased explanatory abilities from the level of below 0.2 to a slightly higher one (0.2 < *q*3 < 0.4). Meanwhile, there were also larger proportions within Grade 1 (29 pairs, 63.0%) and Grade 2 (31 pairs, 50.0%) observed for their moderate enhancements of explanatory abilities from 0.2–0.4 and 0.4–0.6 to 0.4–0.6 and 0.6–0.8, respectively. In other words, the explanatory abilities of potential factors were more likely to be moderately enhanced to higher levels during the pairwise interaction.

**Table 5:** The numbers and *sq* values of 153 pairwise interactions in the nine subgroups

		Group 1	Group 2	Group 3	Group 4	Group 5	
Grade 1	Counts of pairs	1	40	4	\	\	45
	*sq* (%)	159.7	49.3–283.9	159.1–311.6	\	\	49.3–311.6
Grade 2	Counts of pairs	\	15	29	2	\	46
	*sq*(%)	\	21.4–66.1	17.3–121.2	67.0—145.0	\	21.4–145.0
Grade 3	Counts of pairs	\	\	21	31	10	62
	*sq*(%)	\	\	9.5–31.7	7.3–65.1	42.9–57.7	7.3–65.1
		1	55	54	33	10	153

The *q*3 groups: below 0.2, 0.2–0.4, 0.4–0.6, 0.6–0.8, and beyond 0.8. The Maximum (*q*1, *q*2) grades: below 0.2, 0.2–0.4, and 0.4–0.6. \means that the group has no data

## Discussion

Using the GD and other spatial analysis tools, a series of potential influencing factors—and in particular their pairwise interactions—were clearly identified for the spatial differentiation of the gridded incidence of TB in 2017 in the four central districts of Guangzhou, from which several notable findings were obtained. This study provides useful clues for local authorities designing targeted intervention measures to control this disease in Guangzhou and similar municipal regions of China.

Occupational difference of the cases was an obvious characteristic of the 2017 incidence of TB. It has been reported that farmers and workers accounted for the largest proportion of TB cases in some regions of China (e.g., north-east Yunnan Province and Xi'an City) [34–36]. On the contrary, these occupations did not rank first in some highly urbanized regions (e.g., Guangzhou and Foshan), while other occupations (i.e., household and unemployed) were relatively common [37, 38], which was also observed in the central districts of Guangzhou for patients occupied with the household or unemployed (33.1%), the retired population (23.9%), and patients working in the business service (15.1%). However, the TB cases in the study area were distinguishingly featured by their higher percentages of retired patients (61.5%), patients working in business (56.5%) or the catering service (47.3%), students (43.2%), and people working for the cadre (42.7%) in the corresponding occupations of the entire city, which may be attributed to their regional functions (e.g., residential, commercial, educational, and service) [8, 27]. Meanwhile, the study area was also characterized by its slightly higher percentages of TB cases in the > 60-year-old group (34.5%) across the entire city, due to the increasingly aging population [39, 40]. It can thus be seen that the TB cases in the study area possessed their own unique epidemiological characteristics in addition to those shared with cases across the entire city. Accordingly, these TB epidemic features should be considered to design regional appropriate intervention measures (e.g., adequate propaganda and education for these specific populations) to control this disease across the four central districts.

Previous studies have already pointed out that the dominant influencing factors on the distribution of infectious diseases tend to be different due to the varying research units [41, 42]. In our study, the 2 km × 2 km grid was chosen as the appropriate spatial scale on which the gridded TB incidence was spatially clustered, especially in the western part of Tianhe District and the junction area between the Haizhu, Liwan, and Yuexiu districts, owing to their grids having higher incidence. Moreover, the spatial relationship between the gridded TB incidence and most of the selected factors was also easily observed, so that the potential influences on the spatial distribution of the 2017 incidence of TB were sufficiently detected to identify the specific relevant urban units in this study area. The choice of an appropriate spatial scale is essential for identifying the spatial distribution of the incidence of TB and its influencing factors in the target region.

Local TB incidence is often determined by socioeconomic factors, such as the population at risk of spreading this disease, the density and mobility of the population, the transportation system, economic status of the region, and the medical service level on fine scales [7, 12, 16, 17]. Similar findings were obtained in our study: three socioeconomic variables (the 2016 incidence of TB, the counts of officially appointed medical institutions, and the number of bus stops) posed relatively large impacts on the spatial differentiation of the 2017 incidence of TB across the central region of Guangzhou. There was a four-fold increase in transmission risk from some TB patients to their close contacts, causing there to be a high exposure of the susceptible population [9], which may be a reasonable explanation for the strong effects of the 2016 incidence of TB. Another possible interpretation is that the recurrence of previous TB cases after treatment due to the increasing drug resistance of *M. tuberculosis* was very likely to increase the risk of transmission of TB in the regions with high incidence rates in the previous year [43, 44]. As far as the count of officially appointed medical institutions is concerned, its heavy influence on the TB epidemic was probably correlated with medical institutions being representative places where various patients aggregate to ask for health services, including potential TB patients and susceptible people with low immunity [28]. In addition, the number of bus stops was another non-negligible influencing factor for the TB epidemic because both the contact probability among individuals and the population mobility tended to be increased by a convenient public transportation system [17]. In general, the potential variables included in this study could reasonably be the dominant factors influencing the TB epidemic in the study area. Therefore, we cautiously suggest that: (i) the treatment of current TB cases, together with more effective methods dealing with the drug resistance, needs to be considered first to reduce their potential impacts on the incidence of TB during the next year; and (ii) more resources should be rationally allocated to reduce hospital infections and reinforce the propaganda and education for the individuals who often visit the hospital or take the bus.

In comparison to the individual variables, their explanatory abilities were strongly enhanced by their pairwise interactions [45, 46]. Rasam et al. and Ge et al. demonstrated that the interactions between public transportation condition, population density, and urban functional zones had much higher explanatory abilities for the TB epidemic’s spatial differentiations than each individual factor [16, 17]. Our study obtained similar findings; the individual explanatory abilities for the spatial differentiation of the incidence of TB in 2017 across the central region of Guangzhou were remarkably enhanced because of the pairwise interactions. In particular, the contributions of relatively weaker variables (*q* < 0.2) had been significantly enhanced while interacting with bus stops, officially appointed hospitals (i.e., Hosp, Hosp11, and Hosp12), and the 2016 incidence of TB. Among these individual factors, UV, termed for the widely distributed units with crowded population in the low buildings clustered in the study area [29], is a typical urban unit impacting the transmission of *M. tuberculosis* and TB infection [3, 47]. In general, the pairwise interactions made great contributions for interpreting the spatial differentiation of the 2017 incidence of TB across the four central districts. We strongly recommend that the regions with relatively weaker factors should be considered as targets in the prevention and control system, and that comprehensive intervention measures ought to be meticulously implemented in the regions with these paired factors in order to control TB in Guangzhou.

A few limitations should be mentioned here. First, although public transportation defined by bus stops and subway stations was included in this study, population mobility was not adequately considered due to the difficulty of collecting information about population flows, which might be addressed in the future through obtaining and processing either cell phone data or public transportation smart cards. Second, owing to the difficulty of collecting detailed population data with age structure, age-standardized incidence data were not calculated at the gridded scales, which could be possibly resolved through collecting detailed enough population data from the community- and building-based census data. Third, some potential variables related to health services (e.g., constant TB screening for community residents, household surveys, supervision and direction of anti-TB drugs, and follow-up visits for TB patients) supplied by multilevel medical institutions (i.e., township, street, village, and even community level) were not included because of the difficulty of directly calculating the gridded health services’ disparities in this study, which may be resolved in the future through quantifying health service supplies from the perspective of TB patients at various gridded levels because these variables have important effects on this disease. Finally, the TB case data over 10-year or longer periods should be obtained in the future so as to further consolidate and extend the current findings, which are only based on one year’s data.

## Conclusions

A series of socioeconomic and environmental factors, together with their pairwise interactions, were identified as specific urban elements posing important impacts on the spatial differentiations of the gridded TB incidence across the four central districts of Guangzhou. We accordingly suggest that more attention should be paid to the zones with pairwise interactions of these influencing factors in Guangzhou. This study provides meaningful clues for local health authorities designing and implementing effective targeted intervention measures to control this disease in China’s municipal areas, defined by both high urbanization and severe TB epidemics.

## Supplementary Information

**Additional file 1: Fig. S1. **Spatial distribution of the original independent and dependent variables in this study.

## Data Availability

The datasets used and analyzed during the current study are available from the corresponding author on reasonable request.

## Abbreviations

TB: Tuberculosis
Incid-2017: TB incidence in 2017
Incid-2016: TB incidence in 2016
Pop: Population density
GDP: Gross National Product per capita
Hosp: Counts of officially appointed medical institutions
Hosp11: Outpatient hospitals of the officially appointed medical institutions
Hosp12: Inpatient and outpatient hospitals of the officially appointed medical institutions
Road_net: Road network density
Subway: Counts of subway stations
Bus: Counts of bus stops
Residential: Percentage of residential land area
Commercial: Percentage of commercial service land
Pub-serv: Percentage of land for public services
UV: Percentage of urban village area
NDVI: Annual monthly average normalized differential vegetation index
PM_2.5_: Annual monthly average PM_2.5_ concentration
Temp: Average temperature from March to June
Prec: Average precipitation from March to June
Humi: Average humidity from March to June
*r*: The Pearson correlation coefficient
