# Supplementary material for: Specific urban units identified in tuberculosis epidemic using a geographical detector in Guangzhou, China
Source: Infect Dis Poverty. 2022 Apr 15;11:44. doi: 10.1186/s40249-022-00967-z (PMC9012046; doi:10.1186/s40249-022-00967-z)
Supplement: Supplementary file 1 — Additional file 1: Fig. S1. Spatial distribution of the original independent and dependent variables in this study. [file 40249_2022_967_MOESM1_ESM.docx]

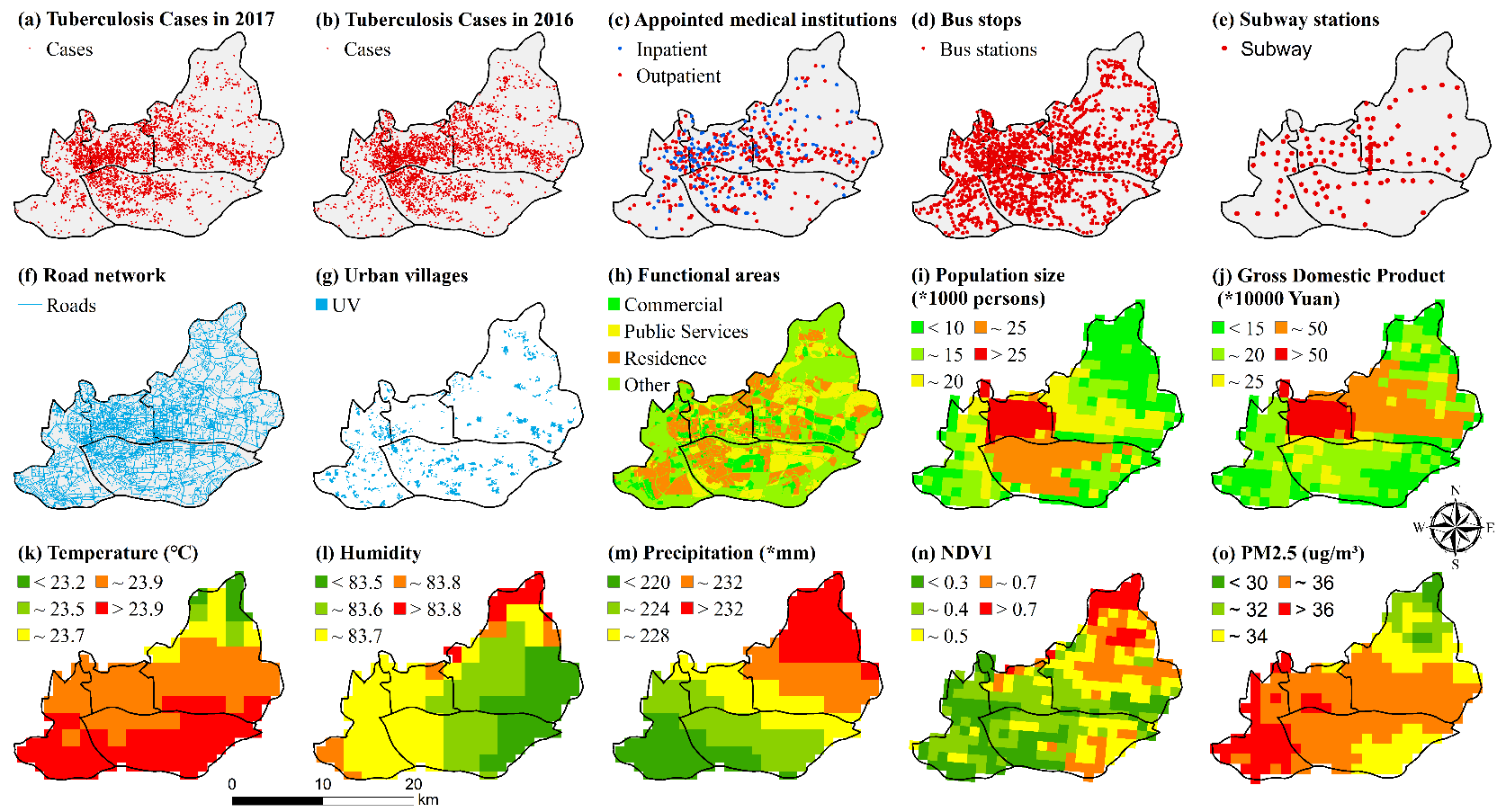


**Fig. S1 Spatial distribution of the original independent (a) and dependent variables (b-o) in this study**
